# Dataset on soil nematode abundance and composition from invaded and non-invaded grassland and forest ecosystems in Europe

**DOI:** 10.1016/j.dib.2024.111098

**Published:** 2024-11-04

**Authors:** Andrea Čerevková, Volodimir Sarabeev, Marek Renčo

**Affiliations:** a Institute of Parasitology, Slovak Academy of Sciences, Hlinkova 3, 04001 Košice, Slovak Republic; b Department of Biology, Zaporizhzhia National University, Zhukovskogo 66, 69063 Zaporizhzhia, Ukraine

**Keywords:** Soil nematodes, Invasive plants, Ecology, Soil health, Grassland, Forest

## Abstract

The dataset presents comprehensive information on soil nematode genera distribution in ecosystems across Slovakia, Poland, Lithuania, and Russia. Data were collected from invaded plots by invasive plants and non-invaded plots from grasslands, deciduous forests, and coniferous forest ecosystems in diverse geographical regions. Invasive plant species included in this dataset are *Asclepias syriaca, Fallopia japonica, Heracleum mantegazzianum, H. sosnowskyi, Impatiens parviflora* and *Solidago gigantea.* The soil properties such as pH, moisture content, carbon, and nitrogen levels were recorded, providing comprehensive information on soil conditions. The data collection process involved standardized soil sampling techniques across all sites, ensuring consistency and comparability. The dataset offers valuable insights into soil nematode biodiversity dynamics in response to plant species invasions in European ecosystems. Nematode genera were classified according to feeding types and colonizer-persister class. Researchers interested in soil ecology, biodiversity conservation, and invasive species management can use this dataset for various purposes. Potential reuses include comparative analyses of nematode community composition, ecological modelling to predict invasive species impacts and assessments of ecosystem health and resilience.

Specifications Table

**Table:** 

Subject	Agricultural Science: Soil Science; Forestry Biological Science: Biodiversity; Zoology; Plant Science (General)
Specific subject area	Soil nematodes, ecology, biodiversity dynamics, invasive species management
Type of data	Raw data in EXCEL files (.xlsx)
Data collection	Nematode genera were isolated from fresh soil samples. Standardized soil sampling techniques were employed across invaded and non-invaded plots from grasslands, deciduous forests, and coniferous forests in Slovakia, Poland, Lithuania, and Russia. Soil samples were collected using soil cores or hand spade, and nematode genus-level identification was performed through morphological identification. The data encompassed 111 taxa of nematodes identified at the genus level. We supply taxa composition and abundance data of each taxon per sample as estimated per 100 g of dry soil. The database also contains information on plant species composition in each sample and soil characteristics such as pH, moisture content, carbon, and nitrogen levels, where available. Invasive plant species presence and habitat characteristics were documented alongside nematode data.
Data source location	The data on presence of soil nematode genera and their abundance and richness were collected from three ecosystems across four Eastern European countries. Grassland **Slovakia**, 1) Košická Belá (G_KB) 48.796572, 21.124076 (10 samples locations); 2) Krásna nad Hornádom (G_KH) 48.668735, 21.306399 (10 samples locations); 3) Opátka (G_Op) 48.802095, 21.057976 (20 samples locations); 4) Vojany (G_Vo) 48.573909, 21.975957 (10 samples locations); 5) Selešťany (G_Se) 48.089196, 19.339782 (10 samples locations); 6) Lekárovce (G_Le) 48.606667, 22.133845 (10 samples locations); 7) Stará Ľubovňa (G_SL) 49.300, 20.700 (40 samples locations); 8) Humenné (G_HE) 49.300014, 20.8020528 (40 samples locations); 9) Rožňava (G_RV) 49.502369, 20.392336 (40 samples locations); **Lithuania**, 10) Kaunas (G_Ka) 54.903000, 23.832000 (16 samples locations); **Poland**, 11) Siechnice (G_Si) 51.038583, 17.158583 (8 samples locations); 12) Czerwonak (G_Cz) 52.46398, 16.98697 (8 samples locations); 13) Mojecice (G_Mo) 51.30149,16.58355 (8 samples locations); **Russia**, 14) Serpukhov district (G_SD) 54.783333, 37.550000 (8 samples locations). Deciduous forest **Slovakia,** 15) Košická Belá (F_D_KB) 48.800586, 21.105234 (10 samples locations); 16) Krásna nad Hornádom (F_D_KH) 48.687847, 21.308521 (10 samples locations); 17) Opátka (F_D_Op) 48.793276, 21.056334 (10 samples locations); 18) Skároš (F_D_Sk) 48.608469, 21.404877 (10 sample s locations); 19) **Lithuania**, Kaunas (F_D_Ka) 54.902000, 23.829000 (8 samples locations); **Russia**, 20) Serpukhov district (F_D_SD) 54.783333, 37.550000 (8 samples locations). Coniferous forest **Slovakia**, 21) Tatranská Lomnica (F_C_TL) 49.176475, 20.284289 (10 samples locations).
Data accessibility	Repository name: Mendeley Data identification number: DOI: 10.17632/6pv5mfh7g8.1 Direct URL to data: https://data.mendeley.com/datasets/6pv5mfh7g8/1

## 1 Value of the Data

• These data compile information on nematode genera distribution across four countries (Slovakia, Poland, Lithuania and Russia) from localities invaded by six alien plant species supplied by samples from control plots across grassland, deciduous and coniferous forest ecosystems, offering a comprehensive view of nematode biodiversity in the Eastern European region. Data on the nematode trophic and colonizer-persister groups was supplied.
• Our data are valuable for researchers in ecology, agriculture, forestry and biodiversity conservation, the dataset expresses nematode population dynamics and their role in ecosystem functioning.
• Researchers can use this dataset to explore biogeographic patterns, assess the impact of environmental factors on nematode communities, and develop predictive models for future ecosystem management.
• The dataset serves as a valuable tool for understanding the relationships between soil organisms - nematodes and their environment affected by plant invasion, thereby contributing to a clearer insight into biodiversity conservation and sustainable land management.

## 2 Background

This is the first attempt to generalize and summarize the results of our ten-year studies on soil nematodes sampled from localities affected by invasive plants [[1], [2], [3], [4], [5], [6], [7], [8], [9], [10], [11], [12]]. We did extensive field surveys across the four European countries to collect nematode samples from various habitats, including grasslands, deciduous forests, and coniferous forests, invaded by six invasive plants (*Asclepias syriaca, Fallopia japonica, Heracleum mantegazzianum, H. sosnowskyi, Impatiens parviflora* and *Solidago gigantea*). Each sample of nematode community from these plants was supplied with data from control plots sampled at the same locality. We used traditional morphological identification using the light microscopy technique to characterize nematode communities at the genus level. The dataset on soil nematodes from both invaded and non-invaded soil ecosystems was created to address gaps in our understanding of soil biodiversity and the effect of alien plants on ecosystem health. Nematodes are ubiquitous organisms in soil ecosystems and play crucial roles in nutrient cycling, decomposition, and trophic interactions.

## 3 Data Description

The dataset comprises one Microsoft® Excel file stored on the Mendeley Data repository.

The Excel file sheet “Nematode Genera” contains raw data on the of nematode genera composition and the abundance of each taxon in soil samples under invasive plants and from control plots. The database covers grasslands, deciduous forests, and coniferous forest ecosystems 16 geographical regions. Invasive plant species included in this dataset are *Asclepias syriaca, Fallopia japonica, Heracleum mantegazzianum, Heracleum sosnowskyi, Impatiens parviflora*, and *Solidago gigantea*.

Soil properties such as pH, moisture content, carbon, and nitrogen levels were recorded (where available), providing detailed information on soil conditions.

The dataset includes the source from which information was retracted (Articles DOI), the country, the study region, geographical coordinates, description of the land habitat, soil type, soil properties, season, month and year of each sample, raw data on the abundance of identified soil nematode genera, their composition and richness value.

The Excel file sheet “Feeding types & c- or p-p class” contain identified nematode genera categorized according to their colonizer-persister (c-p) classification, based on life cycle characteristics. Free-living genera were assigned a c-p score from 1 to 5, while herbivorous genera were assigned a p-p score from 2 to 5 [13]. These nematode genera were also classified by their feeding type and biomass value [14].

## 4 Experimental Design, Materials and Methods

### 4.1 Data collection period and locations

Data were collected from 2013 to 2022 in four countries located in Eastern Europe. The study sites focused on three different ecosystems, including those invaded by invasive plants and control plots. Soil at the sites was classified based on https://soilgrids.org [15].

### 4.2 Soil sampling

Soil samples were collected from a depth of 0–20 cm using either a soil core (⌀ 10 cm) or a hand spade. Each average soil sample was composed of four or five subsamples carefully mixed by hand to create one composite management-specific sample per plot. These composite samples were transferred into plastic bags and taken to the laboratory for processing.

### 4.3 Nematode extraction and identification

Soil nematodes were extracted from 100 g of fresh soil samples using a combination of Cobb sieving and decanting and a modified Baermann technique [16]. The extraction process was conducted over 24 or 48 h. Soil samples were soaked in 1 L of tap water for 60 min to disrupt soil aggregates and promote nematode movement. The soaked sample was carefully passed through a 1-mm sieve (16 mesh) to remove plant parts and debris. Two minutes later, this suspension was passed through a 50-µm sieve (300 mesh) to remove water and very fine soil particles. Nematodes were then extracted from the soil/water suspension using a set of two cotton-polypropylene filters in Baermann funnels at room temperature (approximately 22 °C). Two filter trays were used per sample to limit material thickness to <0.5 cm. Suspensions containing the nematodes were collected after 24 h of extraction. Nematodes were killed in a water bath (approximately 65 °C) and fixed in a 4 % formaldehyde and pure glycerol solution [17].

The total number of individuals per sample was counted using a stereomicroscope (LEICA S8APO, Wentzler Germany, magnification up to 80 ×). Nematode abundance in each sample was expressed as the number of individuals per 100 g of dry soil. The minimum and first 100 individuals' nematodes per sample encountered on slides were identified to genera using a light microscope (Eclipse 90i, Nikon Instruments Europe BV, Amstelveen, Netherlands) at magnifications of 100 ×, 200 ×, 400 ×, 600 × and 1000x, with taxonomic and systematic morphological keys. The data included the composition, abundance, and richness of nematode genera present in the soil samples from both invaded and non-invaded plots.

### 4.4 Soil property determination

A portion of each sample was used to determine soil properties. Soil pH was measured potentiometrically in a water suspension using a digital pH meter, with 10 g of soil mixed with 20 ml of distilled water at room temperature (20 °C). The moisture content of the fresh soil samples (100 g) was estimated gravimetrically by weighing before and after oven drying at 105 °C for 24 h). The total organic C and N were measured by using a Vario MACRO Elemental Analyzer (CNS Version; Elementar, Hanau, Germany).

An exception applies to the data presented in lines 112–143 of Dataset. In these results the moisture content was not measured, therefore, nematode abundance was calculated per 100 g of moist soil. Soil pH was measured in water (1:2.5) and determined using a pH meter (Ekoniks, Moscow, Russia). Total carbon (C) and nitrogen (N) contents were determined by spectrometry (CHN-932, LECO Corp., Saint Joseph, USA) after oxygen combustion at 1100 °C [3].

NA (Not Applicable) marks fields where soil properties were not determined for various reasons.

### 4.5 Nematode feeding types and c-cp/p-p class

Colonizer-persister classification, feeding types and nematode biomass were made by using the online tool Nematode Indicator Joint Analysis (NINJA) (beta) (https://shiny.wur.nl/ninja/) (accessed in August 2024) [14].

## Limitations

Not applicable.

## Ethics Statement

The authors read and followed the ethical requirements for publication in Data in Brief and confirmed that the current work did not involve human subjects, animal experiments, or any data collected from social media platforms.

## CRediT Author Statement

**Andrea Čerevková:** Data collecting, Data curation, Writing, Original draft preparation, **Marek Renčo:** Data collecting, Writing- Reviewing and Editing, **Volodimir Sarabeev:** Conceptualization, Writing- Reviewing and Editing.

## Data Availability

Mendeley DataDatabase on soil nematode abundance and composition from invaded and non-invaded grassland and forest ecosystems in Europe”, Mendeley Data, V1, doi: 10.17632/6pv5mfh7g8.1 (Original data).
